# The Utility of Pre- and Post-Transplant Oral Glucose Tolerance Tests: Identifying Kidney Transplant Recipients With or at Risk of New Onset Diabetes After Transplant

**DOI:** 10.3389/ti.2022.10078

**Published:** 2022-03-17

**Authors:** Julian Singer, Leyla J. Aouad, Kate Wyburn, David M. Gracey, Tracey Ying, Steven J. Chadban

**Affiliations:** ^1^ Department of Renal Medicine, Kidney Centre, Level 2 Professor Marie Bashir Centre, Royal Prince Alfred Hospital, Sydney, NSW, Australia; ^2^ Kidney Node, Charles Perkins Centre, University of Sydney, Sydney, NSW, Australia; ^3^ Central Clinical School, Faculty of Medicine and Health, University of Sydney, Sydney, NSW, Australia

**Keywords:** new onset diabetes after transplant, impaired glucose tolerance, oral glucose tolerance test, kidney transplant, cohort study, NODAT, OGTT, transplant recipients

## Abstract

**Background:** New onset diabetes after transplant (NODAT) is common in kidney transplant recipients (KTRs). Identifying patients at risk prior to transplant may enable strategies to mitigate NODAT, with a pre-transplant oral glucose tolerance test (OGTT) suggested by the KDIGO 2020 Guidelines for this purpose.

**Methods:** We investigated the utility of pre- and post-transplant OGTTs to stratify risk and diagnose NODAT in a retrospective, single-centre cohort study of all non-diabetic KTRs transplanted between 2003 and 2018.

**Results:** We identified 597 KTRs who performed a pre-transplant OGTT, of which 441 had their post-transplant glycaemic status determined by a clinical diagnosis of NODAT or OGTT. Pre-transplant dysglycaemia was identified in 28% of KTRs and was associated with increasing age (*p* < 0.001), BMI (*p* = 0.03), and peritoneal dialysis (*p* < 0.001). Post-transplant dysglycaemia was common with NODAT and impaired glucose tolerance (IGT) occurring in 143 (32%) and 121 (27%) patients, respectively. Pre-transplant IGT was strongly associated with NODAT development (OR 3.8, *p* < 0.001).

**Conclusion:** A pre-transplant OGTT identified candidates at increased risk of post-transplant dysglycaemia and NODAT, as diagnosed by an OGTT. Robust prospective trials are needed to determine whether various interventions can reduce post-transplant risk for candidates with an abnormal pre-transplant OGTT.

## Graphical Abstract

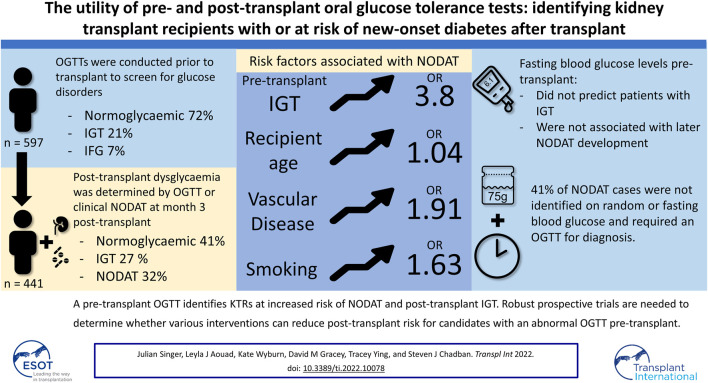

## Introduction

New onset diabetes after transplant (NODAT) occurs commonly following kidney transplantation and is associated with an increase in recipient morbidity and mortality, primarily through the development of cardiovascular disease (1–5). As older age and obesity are becoming more prevalent among kidney transplant candidates and recipient populations over time (6–9), the frequency of NODAT is likely to increase. Identifying patients at risk for NODAT prior to transplantation is therefore of importance to both clinicians and kidney transplant recipients (KTRs). Early recognition of patients at risk for NODAT prior to kidney transplantation may allow for informed risk counselling, a tailored approach towards immunosuppression, and the implementation of targeted interventions to address modifiable risk-factors before and after transplantation.

Abnormalities of glucose metabolism prior to transplant have been shown to predispose recipients to the development of NODAT, although consensus is lacking over which glycaemic parameters are best measured to assess this risk. In general populations, patterns of oral glucose tolerance test (OGTT) results are predictive of future progression to diabetes (10, 11). In kidney transplant candidates, small studies have suggested that random or fasting blood glucose levels may identify patients at risk (12), although larger studies have not borne this out. Stronger evidence supports the role of a pre-transplant OGTT in identifying patients at risk for NODAT, with patients exhibiting impaired glucose tolerance (IGT) following a glucose challenge incurring greater risk (13–16). However, studies to date have been limited by small sample sizes, cyclosporine-based immunosuppression, restriction to recipients from living donors, and variable diagnostic criteria for NODAT (15, 16).

Similarly, current guidelines suggest a number of glycaemic parameters including fasting plasma glucose (FPG), HbA1c, and OGTT to be suitable tests for the detection of diabetes post-transplant (17). Whilst an OGTT remains the gold-standard, practical and economic limitations may constrain its use, leading many centres to rely on FPG alone to screen at risk recipients. However, the performance of FPG as a tool to screen for diabetes post-transplant remains questionable (18).

In this single centre study from a metropolitan transplant referral hospital, we used routine OGTTs to prospectively determine the glycaemic status of kidney transplant recipients prior to and following transplantation between 2003 and 2018. Records were linked to the ANZDATA registry to obtain recipient factors and transplant outcomes. We hypothesised that OGTTs performed prior to and following kidney transplant would outperform FPG in identifying at-risk transplant candidates and KTRs with NODAT, respectively.

## Materials and Methods

### Study Population and Setting

This single centre retrospective cohort study included all non-diabetic adult kidney transplant recipients transplanted at Royal Prince Alfred Hospital, Sydney, Australia, between 1st January 2003 and 31st March 2018. Patients with a diagnosis of diabetes prior to transplant, recipients of combined organ transplants (kidney and liver), patients with a functioning renal allograft *in situ*, and permanent residents of overseas territories were excluded (19).

Results of pre- and post-transplant 2-h 75-g OGTT were obtained from the hospital Electronic Medical Record, the Departmental Database, and patient files.

The deidentified dataset was linked to the ANZDATA registry using deterministic record linkage (transplant centre, date of birth, date of transplant, and sex) to obtain recipient factors including ethnicity, primary kidney disease, history of prior kidney transplants, smoking history, weight, and comorbidities present at time of transplantation (coronary artery disease, peripheral vascular disease, diabetes mellitus, cerebrovascular disease, and chronic lung disease); and transplant characteristics including donor type, donor age, ischaemia time, HLA mismatch, delayed graft function, induction therapy, and transplant outcomes.

ANZDATA is a bi-national registry that collects demographic and kidney-related treatment and outcomes data for all dialysis and transplant patients within Australia and New Zealand. Data is provided on a yearly and voluntary basis by nephrology units with an opt-out system of consent. ANZDATA collection methods and validity have been previously described (20).

The study was conducted following approval by the institutional ethics committee under protocol 2019/ETH06370.

### Oral Glucose Tolerance Testing, Dysglycaemia, and New Onset Diabetes After Transplant

A 75-g OGTT was performed pre- and post-transplant for each participant, conducted according to American Diabetes Association (ADA) guidelines. On the basis of fasting plasma glucose (FPG) and 2-h plasma glucose (2hPG) levels, patients were categorised as having pre-transplant normoglycaemia (FPG <5.6 mmol/L and 2hPG <7.8 mmol/L), impaired fasting glucose (IFG, FPG ≥5.6 mmol/L to 6.9 mmol/L), or impaired glucose tolerance (IGT, 2hPG ≥7.8 mmol/L to 11.0 mmol/L). Patients with a new diagnosis of diabetes (FPG ≥7, or 2hPG ≥11.1) based on their pre-transplant OGTT were excluded from the primary analysis.

The glycaemic status of KTRs was censored at week 12 post-transplant. NODAT was determined by either a positive OGTT result (FPG ≥7, or 2hPG ≥11.1) performed at weeks 10–12 post-transplant, or by a clinical diagnosis defined as repeated elevations in fasting (≥7.0 mmol/L) or random/post-prandial (≥11.0 mmol/L) blood glucose levels throughout the post-transplant period that required ongoing treatment with antidiabetic medication at week 12 post-transplant. Patients not requiring antidiabetic medication and for whom the results of a 75g OGTT were not attainable were classified as having an unknown glycaemic state due to insufficient evaluation.

### Statistical Analysis

Data in the manuscript are expressed as means ± standard deviation for normally distributed data or median ± interquartile range for non-normally distributed data, and as frequencies for categorical variables.

Differences in continuous variables between groups were examined by analysis of variance (ANOVA) for normally distributed data, or by the non-parametric Kruskal-Wallis log rank test for non-normally distributed data. Categorical variables were compared using the Chi squared test. Cohen’s kappa was used to determine the agreement between the fasting and 2-h plasma glucose criteria for NODAT, and the correlation between fasting and subsequent 2-h glucose levels by the Pearson correlation coefficient. Receiver operating characteristic (ROC) curve analysis was conducted to identify the diagnostic utility of FPG value at time of OGTT in identifying pre and post-transplant dysglycaemia.

To ascertain the associations between patient factors and the development of NODAT we performed multivariate analysis using a generalised linear model with a logit link function. Variables were included if they were statistically associated with the outcome by univariate analysis (*p* < 0.1) or selected a priori on the basis of published associations. The results of the model are expressed as crude and adjusted odds ratios (ORs) with 95% confidence intervals (CIs).

Patient and graft survival were analysed by the Kaplan-Meier method and compared using the log-rank test for unadjusted survival, with Cox proportional hazard regression used for multivariate analyses.

For all analyses, a two-sided *p* < 0.05 was considered statistically significant. All statistical analysis was performed using R Statistical Software (2019; R Foundation for Statistical Computing, Vienna, Austria).

### Sensitivity Analyses

As not all kidney transplant recipients at our centre underwent pre-transplant assessment with an OGTT, we conducted a sensitivity analysis to determine whether the association of post-transplant dysglycaemia and transplant outcomes remained consistent when the entire transplant cohort with known glycaemic status post-transplant were examined. This cohort consisted of an additional 114 KTRs who did not undergo pre-transplant assessment with an OGTT but had their post-transplant glycaemic status accurately determined by either a clinical diagnosis of NODAT or the results of an OGTT. A further 197 KTRs who had a pre-transplant diagnosis of diabetes were included as a third comparator.

## Results

### Patient Characteristics

**FIGURE 1:**
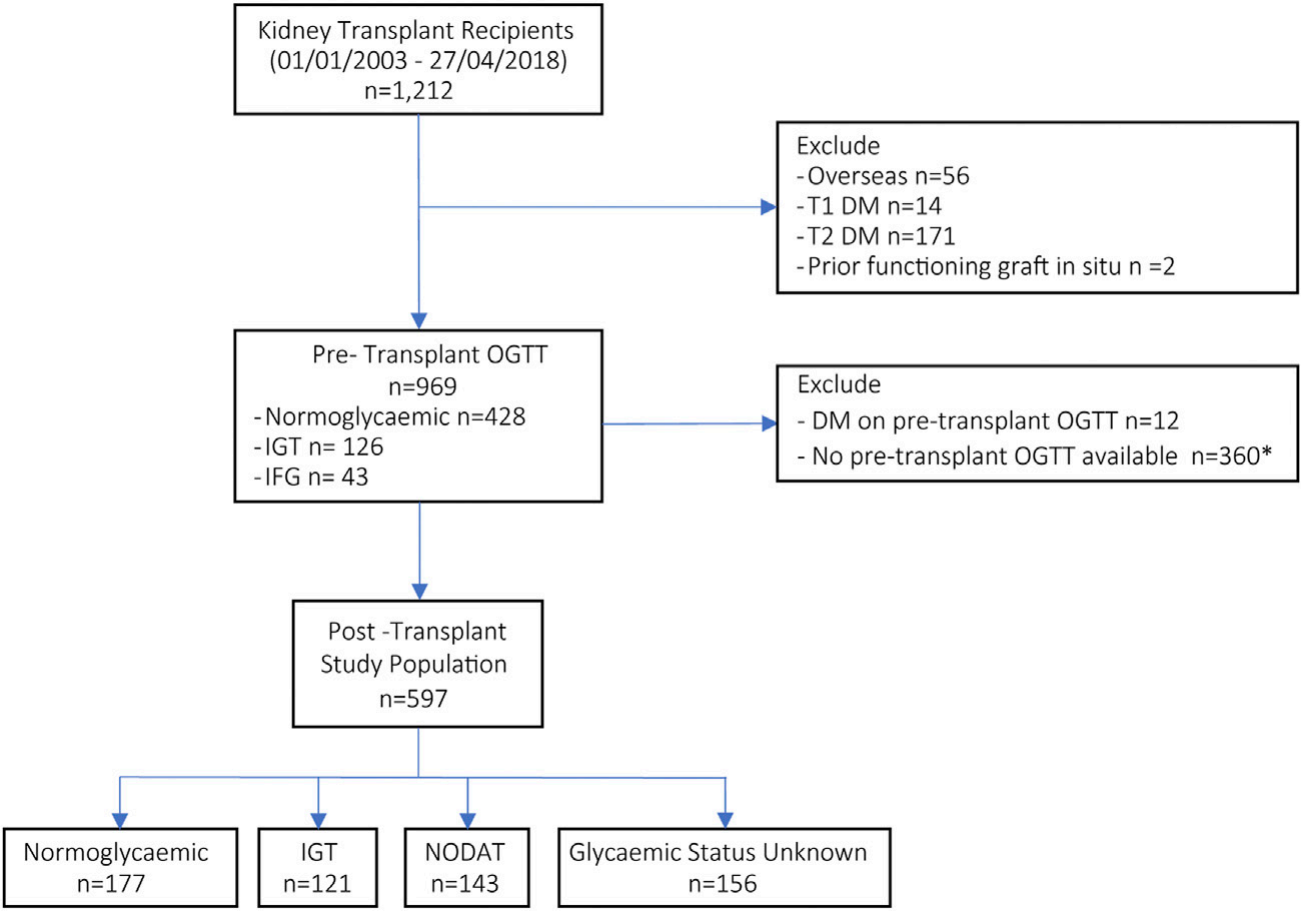
Flow diagram for enrolment and stratification of recipients according to pre- and post-transplant glycaemic status. (*114 transplant recipients who did not perform an OGTT pre-transplant had a known post-transplant glycaemic status and were included in the sensitivity analyses, in addition to 197 recipients with pre-transplant DM.) DM, diabetes mellitus; OGTT, oral glucose tolerance test; IGT, impaired glucose tolerance; IFG, impaired fasting glucose; NODAT, new onset diabetes after transplant.

A total of 1212 kidney only transplants were performed in our centre between January 2003 and the end of April 2018. We excluded 56 transplants performed with recipients whose usual residence was outside of Australia (19), 2 recipients with a prior functioning renal allograft at the time of transplant, and a further 185 patients with a pre-existing diagnosis of diabetes. For the remaining cohort, results of a pre-transplant 75-g OGTT were obtained for 609 recipients, with an additional 12 cases of unrecognised diabetes identified and subsequently excluded from the study (Figure 1).

Baseline characteristics of the final study population (*n* = 597) are shown in Table 1. The mean age of recipients was 47 ± 14, with 64% being male and 73% of white descent. The mean body mass index (BMI) was 26 ± 5 kg/m^2^, and the primary cause of ESKD was glomerulonephritis (49.2%), polycystic kidney disease (15.2%), reflux nephropathy/posterior urethral valves (PUV) (8%), renovascular disease in 8%, and other causes in the remaining 19.4%. 370 (62%) recipients were receiving maintenance haemodialysis prior to transplantation, 136 (22.8%) peritoneal dialysis, and 91 (15.2%) were pre-emptively transplanted before commencing dialysis. Donor organs were received from living (*n* = 297) or deceased (*n* = 300) donors.

**TABLE 1:** Characteristics of kidney transplant recipients stratified by post-transplant glycaemic status.

	Normoglycaemic	IGT	NODAT	Unknown	p
	*n* = 177	*n* = 121	*n* = 143	*n* = 156	
Age (mean (SD))	41.5 ± 13.5	49.7 ± 12.8)	53.9 ± 11.4	47.7 ± 14.2	<0.001
Age ≥ 50 (%)	49 (27.7)	69 (57.0)	94 (65.7)	70 (44.9)	<0.001
Gender					0.967
Male (%)	116 (65.5)	76 (62.8)	91 (63.6)	102 (65.4)	
Female (%)	61 (34.5)	45 (37.2)	52 (36.4)	54 (34.6)	
BMI (mean (SD))	25.4 ± 5.5	26.3 ± 4.6	26.2 ± 4.5	26.3 ± 4.9	0.326
BMI Category (%)					0.593
Underweight (<18.5)	9 (5.1)	4 (3.3)	5 (3.5)	6 (3.8)	
Normal (≥18.5 to <25.0)	84 (47.5)	45 (37.2)	58 (40.6)	57 (36.5)	
Overweight (≥25.0 to <30.0)	52 (29.4)	45 (37.2)	46 (32.2)	52 (33.3)	
Obese (≥30)	27 (15.3)	25 (20.7)	31 (21.7)	34 (21.8)	
Not Available	5 (2.8)	2 (1.7)	3 (2.1)	7 (4.5)	
Racial Background (%)					0.243
Caucasian	129 (72.9)	93 (76.9)	99 (69.2)	116 (74.4)	
Asian	32 (18.1)	21 (17.4)	33 (23.1)	17 (10.9)	
Aboriginal/Torres Strait Islander	1 (0.6)	1 (0.8)	4 (2.8)	5 (3.2)	
Other	15 (8.4)	6 (5.0)	7 (4.9)	18 (11.5)	
Primary Renal Disease (%)					0.068
Glomerulonephritis	93 (52.5)	67 (55.4)	69 (48.3)	65 (41.7)	
Polycystic Kidney Disease	22 (12.4)	23 (19.0)	20 (14.0)	26 (16.7)	
Reflux Nephropathy/PUV	13 (7.3)	8 (6.6)	9 (6.3)	18 (11.5)	
Hypertension	17 (9.6)	4 (3.3)	18 (12.6)	9 (5.8)	
Other	32 (18.1)	19 (15.7)	27 (18.9)	38 (24.4)	
RRT Prior To Transplant (%)					0.327
Haemodialysis	109 (61.6)	68 (56.2)	88 (61.5)	105 (67.3)	
Peritoneal	34 (19.2)	32 (26.4)	38 (26.6)	32 (20.5)	
Pre-emptive transplant	34 (19.2)	21 (17.4)	17 (11.9)	19 (12.2)	
Living Donor (%)	110 (62.1)	65 (53.7)	67 (46.9)	55 (35.3)	<0.001
Prior Kidney Transplant (%)	18 (10.2)	8 (6.6)	11 (7.7)	25 (16.0)	0.055
Smoking History (%)	48 (27.0)	36 (29.8)	62 (43.3)	70 (44.9)	0.001
Prior Vascular Disease^a^ (%)	28 (15.8)	21 (17.4)	45 (31.5)	26 (16.7)	0.002
Induction Immunosuppression					
IL-2 Receptor antibody (%)	149 (84.2)	109 (90.1)	124 (86.7)	114 (73.1)	0.001
T cell depleting antibody (%)	7 (4.0.)	5 (4.1)	4 (2.8)	6 (3.8)	0.931
B cell depleting antibody (%)	4 (2.3)	2 (1.7)	3 (2.1)	0 (0.0)	0.335
Intravenous Immunoglobulin (%)	17 (9.6)	13 (10.7)	15 (10.5)	19 (12.2)	0.937
Maintenance Immunosuppression					
Tacrolimus v CSA (%)	152 (88.4)	97 (80.8)	127 (89.4)	148 (95.5)	0.002
CNI Free (%)	5 (2.8)	1 (0.8)	1 (0.7)	1 (0.6)	0.242
mTOR (%)	49 (27.7)	26 (21.5)	34 (23.8)	11 (7.1)	<0.001
Prednisolone (%)	177 (100.0)	121 (100.0)	143 (100.0)	155 (99.4)	0.416
- Dose (mg) at 3 m (mean, SD)	11.1 ± 5.5	10.7 ± 2.9	11.5 ± 8.1	11.1 ± 3.6	0.790
HLA MM (%)					0.068
1–2	63 (35.6)	44 (36.4)	48 (33.6)	46 (29.5)	
3–4	70 (39.5)	41 (33.9)	41 (28.7)	47 (30.1)	
5–6	44 (24.9)	36 (29.8)	54 (37.8)	63 (40.4)	
Rejection episode (any) (%)	38 (21.5)	25 (20.7)	32 (22.4)	37 (23.7)	0.931
Early rejection (≤ 90 days post-transplant) (%)	26 (14.7)	15 (12.4)	29 (20.3)	32 (20.5)	0.179
Delayed graft function (%)	20 (11.3)	17 (14.0)	25 (17.5)	38 (24.4)	0.011
eGFR (CKD-EPI)					
at 3 m (mean, SD)	55.9 ± 18.5	53.1 ± 18.1	51.3 ± 16.5	48.7 ± 17.5	0.004
at 1 year (mean, SD)	60.2 ± 18.8	52.2 ± 15.4	52.6 ± 18.6	51.2 ± 18.5	<0.001

a Coronary artery disease, peripheral vascular disease, or cerebrovascular disease.

The majority of patients received induction with intravenous methylprednisolone and basiliximab (83%), with antithymocyte induction (3.7%) and/or intravenous immunoglobulin (10.7%) administered to higher-immunologic risk recipients. Initial immunosuppression was with tacrolimus (89%) or cyclosporine (10%), mycophenolate (98%) and/or sirolimus/everolimus (20%); and all except one recipient received maintenance prednisolone. Tacrolimus trough concentrations of 10–12 ng/ml were targeted during the first 3 months post-transplant, and 5–8 ng/ml from month 3 onward depending on immunological risk.

Pre-transplant OGTTs were performed at a median of 367 (IQR: 166–714) days prior to transplantation. Dysglycaemia determined by OGTT before transplantation was common, affecting 27% of the cohort, with IGT (126, 21%) more prevalent than IFG (43, 7%); the remaining 428 tests (72%) were normal (Table 2).

**TABLE 2:** Results of oral glucose tolerance tests performed prior to and following kidney transplantation, stratified by post-transplant glycaemic status.

	Normoglycaemic	IGT	NODAT	Unknown	p
	*n* = 177	*n* = 121	*n* = 143	*n* = 156	
Pre-Transplant OGTT					
Day pre-transplant (median [IQR])	−282 [−551, −146]	−407 [−746, −211]	−367 [−672, −142]	−440 [−736, −227]	0.002
FPG mmol/L [mean (SD)]	4.77 (0.49)	5.07 (0.59)	5.07 (0.73)	4.81 (0.56)	<0.001
2hPG mmol/L [mean (SD)]	5.58 (1.49)	6.54 (1.60)	7.37 (1.90)	5.98 (1.80)	<0.001
Glycaemic status pre-transplant					<0.001
Normoglycaemic (%)	151 (85.3)	81 (70.0)	74 (51.7)	122 (78.2)	
IFG (%)	9 (5.1)	16 (13.2)	9 (6.3)	9 (5.8)	
IGT (%)	17 (9.6)	24 (19.8)	60 (42.0)	25 (16.0)	
Post-Transplant OGTT					
Day post-transplant (median [IQR])	77 [68, 92]	72 [69, 91]	73 [65, 88]	—	0.312
FPG mmol/L (mean (SD))	4.95 (0.46)	5.24 (0.58)	5.76 (0.89)	—	<0.001
2hPG mmol/L (mean (SD))	6.21 (1.12)	9.14 (0.95)	13.08 (2.20)	—	<0.001

OGTT, oral glucose tolerance test; IGT, impaired glucose tolerance; IFG, impaired fasting glucose; NODAT, new onset diabetes after transplant.

Patients with pre-transplant dysglycaemia (IGT or IFG) were older (52 ± 12 years vs. 45 ± 14 years, *p* < 0.001), had a higher BMI (27 ± 5 m/kg^2^ vs. 26 ± 5 m/kg^2^, *p* = 0.03), and were more commonly undergoing peritoneal dialysis (35.3% vs. 17.7%, *p* < 0.001). The association with peritoneal dialysis was largely driven by a higher prevalence of IFG, potentially relating to glucose absorption from dialysate (Supplementary Table S1).

### Pre-Transplant FPG and Prediction of IGT

**FIGURE 2:**
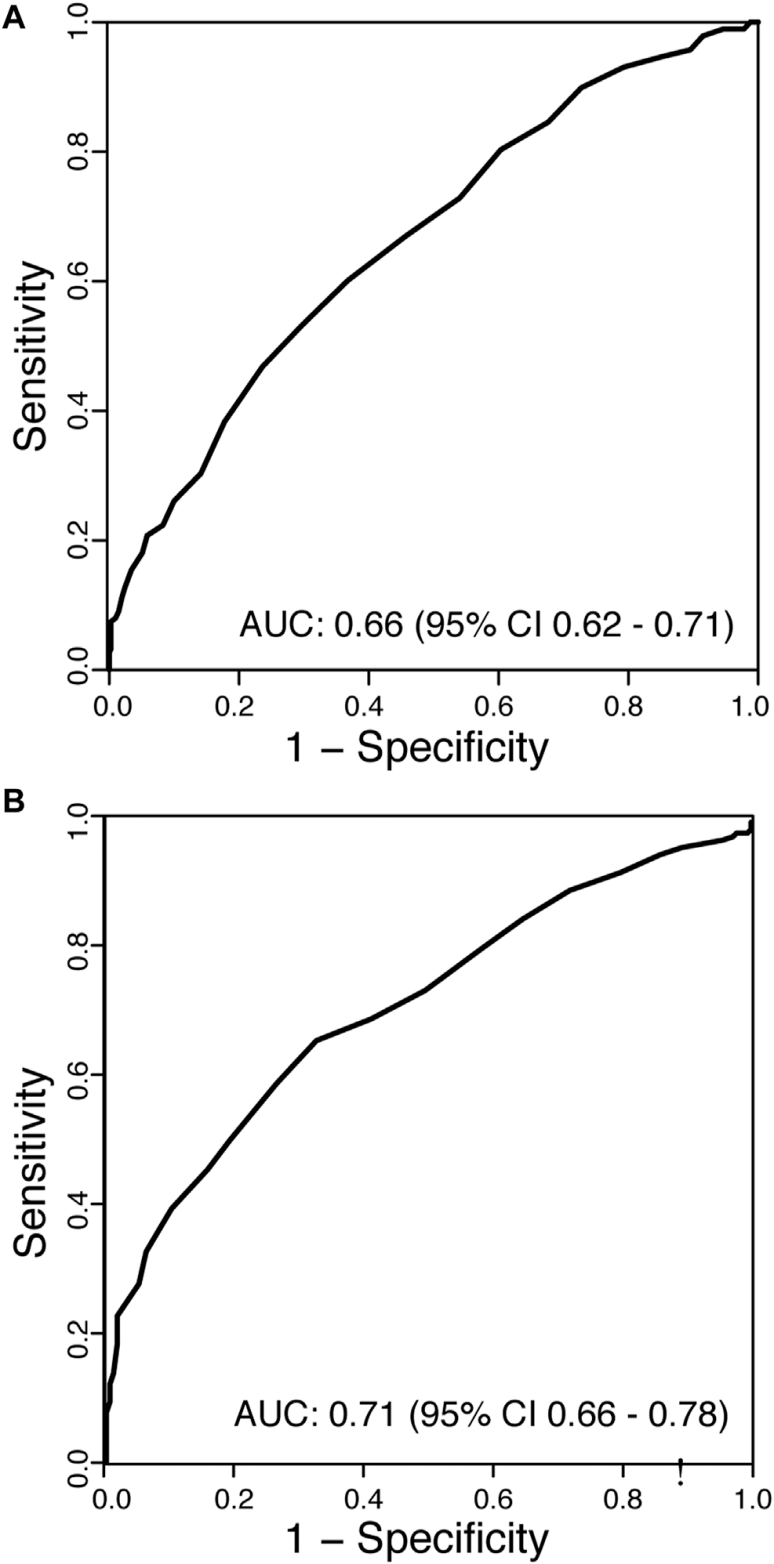
Receiver operating characteristic (ROC) curves for **(A)** fasting plasma glucose (FPG) predicting impaired glucose tolerance (IGT) in patients pre-transplant, and **(B)** FPG predicting dysglycaemia (NODAT or IGT) post-transplant. AUC, area under the curve.

As elevated FPG levels have been advocated as a screening test to identify patients who would benefit from further investigation with an OGTT pre-transplant, we examined the predictive value of this approach. The mean FPG of patients in the normoglycaemic group was 4.8 ± 0.5 mmol/L compared to 5.2 ± 0.7 mmol/L in those with IGT (*p* < 0.001). However, FPG values only weakly correlated with subsequent 2hPG levels (*r* = 0.36, *p* < 0.001). The receiver operating characteristic (ROC) curve for FPG predicting an abnormal OGTT is shown in Figure 2A. The AUC was 0.66 (95% CI: 0.62–0.71), suggesting that FPG has little value in identifying patients who would be found to have IGT or diabetes by OGTT pre-transplant. Table 3 displays the test characteristics for FPG cut-off values predictive of IGT pre-transplant, identifying a FPG of 5.05 mmol/L as having the optimal test performance, but with a sensitivity and specificity of 53% and 70% respectively. Thus, if patients were only selected to undergo an OGTT based on an abnormal FPG reading (≥5.6 mmol/L), 78% of KTRs with pre-transplant dysglycaemia would not be identified.

**TABLE 3:** Fasting plasma glucose cut-off values for the detection of impaired glucose tolerance pre-transplant.

FPG (mmol/L)	Sensitivity (%)	Specificity (%)	FPR	FNR	PPV	NPV	Youden index
4.60	85	32	0.68	0.15	0.36	0.82	1.17
4.80	73	46	0.54	0.27	0.38	0.79	1.19
5.00	60	63	0.37	0.40	0.43	0.78	1.23
5.05	53	70	0.30	0.47	0.45	0.77	1.24
5.20	47	76	0.24	0.53	0.48	0.76	1.23
5.40	30	86	0.14	0.70	0.50	0.73	1.16
5.60	22	92	0.08	0.78	0.55	0.72	1.14
5.80	18	95	0.05	0.82	0.62	0.72	1.13
6.00	13	98	0.02	0.87	0.71	0.71	1.10

FPR, false positive ratio; FNR, false negative ratio; PPV, positive predictive value; NPV, negative predictive value.

### Post-Transplant Glycaemic Status

Of the 597 KTRs assessed, post-transplant glycaemic status could be accurately determined in 441 cases by either a clinical diagnosis of NODAT (*n* = 85), or by the results of an OGTT (*n* = 358) conducted at a median of 74 days post-transplant (IQR: 67–91 days). Disorders of glycaemia were common post-transplant with 143 patients (33%) developing NODAT and a further 121 (28%) displaying IGT. For the remainder, the OGTT was normal (*n* = 159, 37%) or revealed isolated IFG (*n* = 18, 4%).

### Comparison of NODAT Evident by FPG or Post 2-h Glucose Load

Of the 143 KTRs with NODAT, 59 (41%) diagnoses were not established on clinical grounds and were detected by protocolised OGTT at 10 weeks post-transplant. Whilst all 59 patients met ADA diagnostic criteria by an elevated 2hPG, only 3 patients met FPG criteria (FPG ≥7 mmol/L). The concordance between the fasting and 2-h glucose criteria for the diagnosis of NODAT was poor (κ = 0.07).

In patients without clinical NODAT, post-transplant FPG levels were a poor indicator of KTRs likely to have dysglycaemia on formal testing (Figure 2B, AUC 0.71, 95% CI: 0.66–0.78). The optimum decision threshold for an FPG to proceed to an OGTT was 5.15 mmol/L, with a sensitivity and specificity of 66% and 76%, respectively. If the ADA criteria for an abnormal FPG (≥5.6 mmol/L) was applied to identify KTR without clinical NODAT who should undergo an OGTT post-transplant, 60% of KTR with occult dysglycaemia would be missed (Table 4).

**TABLE 4:** Fasting plasma glucose cut-off values for the detection of dysglycaemia (IGT or NODAT) post-transplant.

FPG (mmol/L)	Sensitivity (%)	Specificity (%)	FPR	FNR	PPV	NPV	Youden index
4.60	92	20	0.80	0.08	0.54	0.72	1.12
4.80	85	35	0.65	0.15	0.57	0.70	1.20
5.00	74	51	0.49	0.26	0.60	0.66	1.24
5.15	66	67	0.33	0.34	0.67	0.66	1.33
5.20	66	67	0.33	0.34	0.67	0.66	1.33
5.40	50	81	0.19	0.50	0.73	0.62	1.31
5.60	40	90	0.10	0.60	0.80	0.60	1.30
5.80	28	95	0.05	0.72	0.85	0.57	1.23
6.00	18	98	0.02	0.82	0.92	0.55	1.17

FPR, false positive ratio; FNR, false negative ratio; PPV, positive predictive value; NPV, negative predictive value.

Of our cohort, 156 (26%) patients did not develop clinical NODAT and did not undergo post-transplant OGTT. This group were similar in age (47 ± 14 vs. 46 ± 13, *p* = 0.63) and BMI (26.3 ± 4.9 vs. 25.7 ± 5.1, *p* = 0.246) to those for whom an OGTT was recorded, with similar glucose profiles recorded prior to transplant (FPG 4.8 ± 0.6 mmol/L v 4.9 ± 0.6 mmol/L, *p* = 0.107; and 2hPG 6.0 ± 1.8 mmol/L v 6.1 ± 1.7 mmol/L, *p* = 0.46) (Supplementary Table S2). We conducted multivariate analysis to determine whether this group differed significantly from the cohort with recorded OGTTs (Supplementary Table S3), and found they were more likely to have been referred from and returned to care outside the transplant centre (OR = 2.42, 95% CI: 1.62–3.62, *p* < 0.001), and to have received a kidney from a deceased donor (OR = 2.25, 95% CI: 1.46–3.48, *p* < 0.001).

### Risk Factors for the Development of NODAT

**FIGURE 3:**
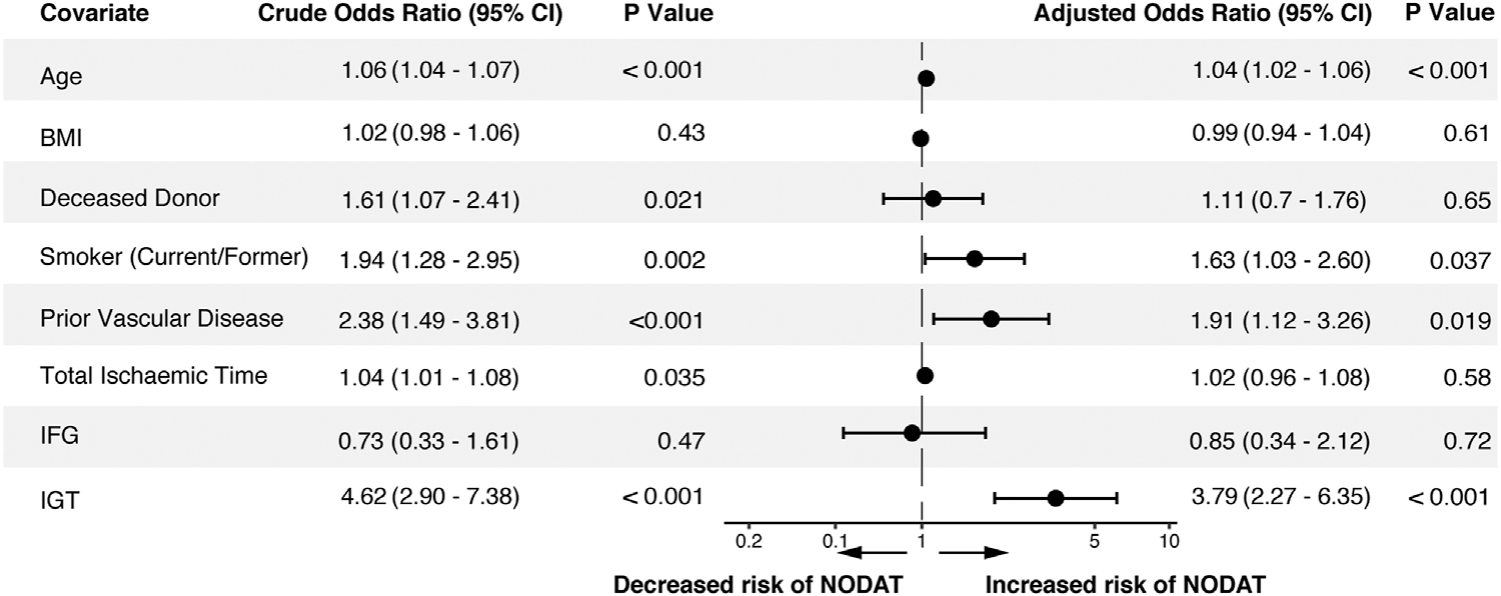
Risk factors for the development of NODAT following univariate and multivariate analysis. BMI, body mass index; IGT, impaired glucose tolerance; IFG, impaired fasting glucose; NODAT, new onset diabetes after transplant.

Covariates associated with the development of NODAT are shown in Figure 3. Patient factors not associated with the development of NODAT by univariate analysis included BMI, gender, primary renal disease, prior kidney transplantation, the type of induction therapy or calcineurin inhibitor used, and the occurrence of rejection within the first 90 days post-transplant. After multivariate analysis, age at transplant remained a significant risk-factor, conferring a 4% increase in risk of NODAT per year of age. Pre-transplant IGT (OR = 3.79, 95% CI: 2.27–6.35, *p* < 0.001), but not IFG, was significantly associated with NODAT (Figure 3).

### Patient and Graft Outcomes

**FIGURE 4:**
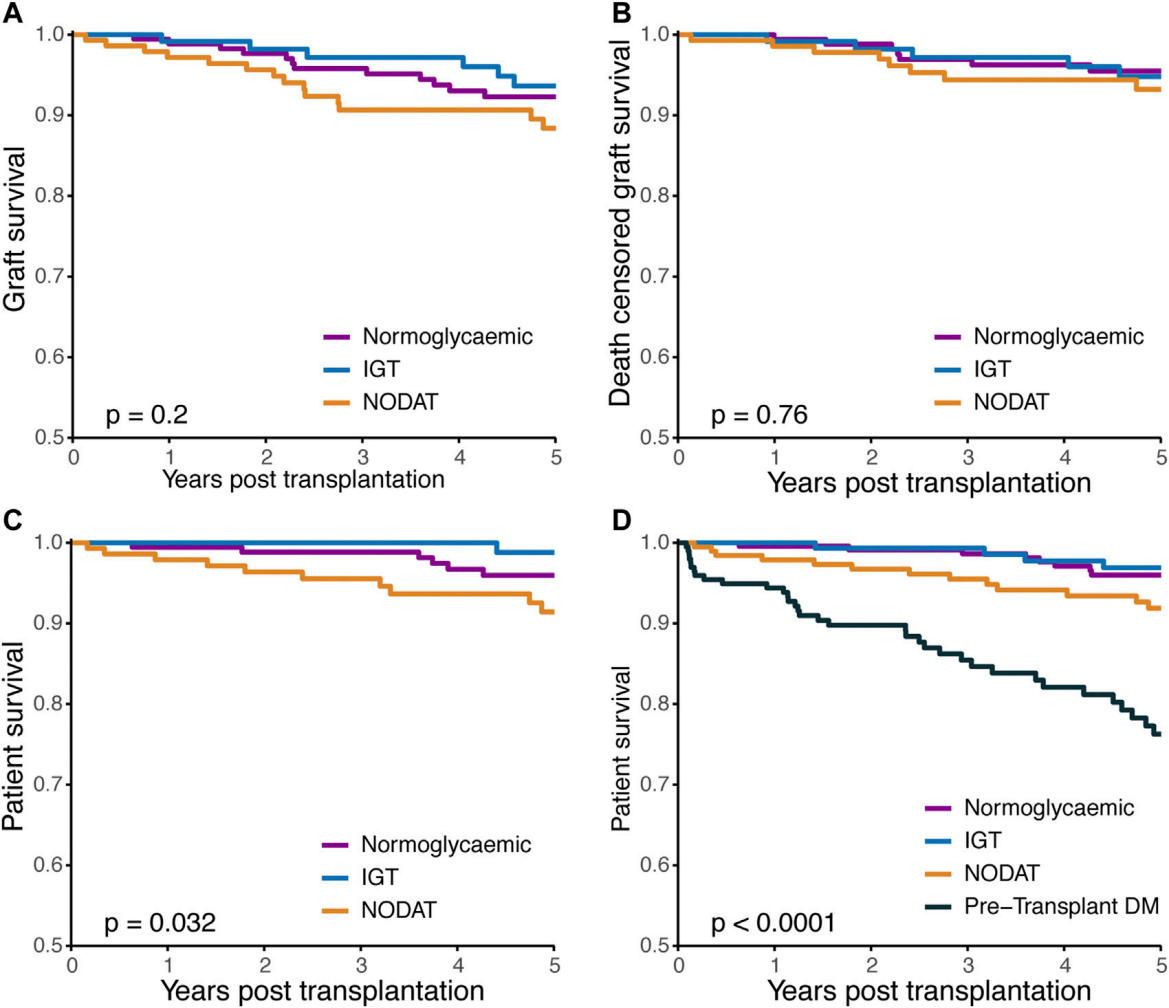
Kaplan-Meier plots of **(A)**, graft survival **(B)**, death censored graft survival, and **(C)**, patient survival according to post-transplant glycaemic status. **(D)** Patient survival of all kidney transplant recipients with a known post-transplant glycaemic status, including KTRs with pre-transplant diabetes (*n* = 781). IGT, impaired glucose tolerance; NODAT, new onset diabetes after transplant; DM, diabetes mellitus.

Kaplan-Meier plots of graft survival, death-censored graft survival, and patient survival are shown in Figure 4. In the cohort of patients with a known glycaemic status post-transplant, graft survival at 5-years was 91% (95% CI: 89–94%) and 95% (95% CI: 92–97%) when censored for death. No significant difference in graft survival was observed between the glycaemic cohorts, without or with censoring for death of the patient (Figures 4A,B, *p* = 0.2 and *p* = 0.76). Patient survival was inferior for patients with NODAT compared to normoglycaemic recipients. (Figures 4C, *p* = 0.032). Whilst patients with NODAT experienced higher rates of mortality compared to normoglycaemic KTRs (HR 2.29, 95% CI: 1.21–4.32, *p* = 0.012), only increasing recipient age (HR 1.03, 95% CI: 1.01–1.06, *p* = 0.034) and a pre-transplant history of vascular disease (HR 2.65, 95% CI: 1.35–5.28, *p* = 0.006) were associated with an increased risk of death in a multivariate analysis (Table 5).

**TABLE 5:** Univariate and multivariate Cox regression analysis of covariates associated with death post-transplant.

	Crude HR (95% CI)	P (Wald’s Test)	Adjusted OR (95%CI)	P (Wald’s Test)
NODAT^a^	2.29 (1.21–4.32)	0.024	1.37 (0.69–2.72)	0.369
Age at transplant	1.05 (1.02–1.08)	<0.001	1.03 (1.01–1.06)	0.034
Deceased donor	2.74 (1.42–5.28)	0.002	1.92 (0.97–3.81)	0.061
Prior vascular disease^b^	3.94 (2.08–7.46)	<0.001	2.65 (1.35–5.28)	0.006

NODAT, new onset diabetes after transplant.

a normoglycaemia as reference group.

b coronary artery disease, peripheral vascular disease, or cerebrovascular disease).

### Sensitivity Analyses

Five-years graft survival of all KTRs transplanted during the study period with a known post-transplant glycaemic status and 182 KTRs previously excluded because of known pre-transplant DM are shown in Figure 4D. The Kaplan-Meier plots reveal a hierarchy of risk for mortality, strongest for pre-transplant DM over NODAT, IGT and normoglycaemia. By multivariate analysis, pre-transplant diabetes (HR 2.77, 95%CI: 1.54–4.98, *p* < 0.001), but not NODAT or IGT, was strongly associated with decreased survival post-transplant.

## Discussion

In a large cohort of KTRs managed with contemporary immunosuppression, an OGTT conducted as part of pre-transplant candidate evaluation revealed unrecognised diabetes in 2% and IGT in 28%. Following transplantation, those with IGT incurred a greater than 3-fold higher incidence of NODAT as compared to their normoglycaemic peers. Elevated fasting glucose pre-transplant was not predictive of NODAT, nor did it identify a subset of candidates likely to manifest IGT or DM pre-transplant. These findings highlight the utility of routine pre-transplant OGTT to identify risk of NODAT, and thereby provide opportunities to recognise, discuss and potentially mitigate the negative impacts of NODAT on post-transplant survival. This data lends support to the 2020 KDIGO Guidelines on the management of Candidates for Kidney Transplantation where evaluation with a pre-transplant OGTT has been suggested for this purpose (21).

A secondary finding of our study was the utility of a protocolised, post-transplant OGTT to diagnose clinically inapparent NODAT and to identify KTRs with IGT. In addition to the 19% of KTRs with clinically apparent NODAT, OGTT detected NODAT in a further 14% yielding a total incidence of 33% in those who underwent thorough assessment. A further 121 KTRs exhibited IGT, thus use of post-transplant OGTT identified clinically unrecognised dysglycaemia in 42% of our cohort. Given the increase in cardiovascular risk associated with NODAT and IGT following kidney transplantation (2, 22), an OGTT is essential in order to identify at risk KTRs and create an opportunity for the implementation of appropriate risk-reduction strategies.

We recognise that widespread uptake of OGTTs has been limited by practical and economic constraints. For this reason, its use as a screening tool in transplant assessment has often been restricted to those with identified risk factors, such as a prior elevated FPG level (21). Our findings suggest that this approach is of little value. We found that pre-transplant FPG levels, in our study taken at the time of an OGTT, correlated poorly with subsequent 2hPG levels. Furthermore, FPG levels were of no discriminatory value in predicting transplant candidates who had IGT, and unlike IGT were not associated with the development of NODAT post-transplant.

The prevalence of pre-transplant dysglycaemia in our cohort is concordant with previously reported rates of IGT amongst kidney transplant candidates (23). These rates are significantly higher than the general, age-matched Australian population (24), and may reflect the increase in basal insulin resistance amongst patients with ESKD (25). The insensitivity of FPG to detect dysglycaemia, coupled with the high incidence of dysglycaemia amongst candidates for kidney transplantation highlights the need for an OGTT to be performed as part of routine candidate assessment.

NODAT occurs commonly in KTRs although the reported incidence varies according to the diagnostic criteria employed, timing post-transplant, and the type of immunosuppression used. At month three post-transplant the incidence of recorded NODAT in our cohort was 24%, consistent with previous studies where protocolised OGTTs have been performed (13, 26, 27). Lower rates have been reported in cohorts which have relied on clinical records or non-dynamic glucose testing (28–30), and higher rates in studies which included dysglycaemia recorded during the early post-transplant period (31).

In this study, 41% of NODAT cases were not identified by routine surveillance of blood glucose levels and were only diagnosed by the use of a screening OGTT. We found FPG to not only lack sufficient sensitivity to identify patients with NODAT, but to poorly predict KTRs who would return an abnormal OGTT. Importantly, as the diagnosis of IGT in KTRs is clinically significant (32, 33) and can only be achieved with an OGTT, our findings suggest that all kidney transplant recipients without clinically evident NODAT, should undergo an OGTT to screen for the presence of occult NODAT or IGT (34).

We confirmed well-known risk factors for NODAT such as increasing age and bring attention to the impact of smoking (35). Interestingly, in our cohort BMI was not associated with the development of dysglycaemia post-transplant. Our study is not alone in presenting this finding (28, 36, 37), which may be due to different demographic populations, the short follow-up time and differences in the diagnostic criteria for NODAT. Populations with a strong association between BMI and NODAT, such as African Americans were not represented in our cohort (3), whilst Asian populations, which contributed to 17% of our cohort are at an increased risk for NODAT despite lower BMIs (38, 39). Other reported risk-factors, such as the type of calcineurin inhibitor (27) and early rejection events did not correlate with post-transplant dysglycaemia, and may be explained by the infrequent occurrence of both cyclosporine use and rejection events in our cohort. As a practice-derived cohort, it is also likely that the finding of dysglycaemia on pre-transplant OGTT may have influenced the choice of calcineurin inhibitor for some patients (40). In contrast to the findings of Caillard et al (13), we did not find ADPKD to be associated with an increased risk of NODAT, despite a similar incidence of ADPKD and NODAT across both cohorts.

The development of NODAT is associated with an increased risk of adverse events, particularly cardiovascular morbidity and mortality (1–3, 41, 42). IGT has also been shown to convey a similarly increased risk of cardiovascular events in both KTRs (2, 22, 43) and general populations (44), however its impact on overall mortality appears less clear (45). Neither NODAT or IGT were independently associated with all-cause mortality in our cohort, and a number of factors may have contributed to these findings. Firstly, we recorded the incidence of dysglycaemia at 3 months post-transplant, and acknowledge that 20–30% of cases may revert to a normoglycaemic state within the first post-transplant year (22, 33). Whilst defining NODAT at an early timepoint may have reduced the sensitivity of our survival analysis, our approach of early NODAT detection is supported by previous studies which have associated early detection (<3 months) with an increased risk of future cardiovascular events and death (5). Secondly, previous studies reporting lower patient survival with NODAT have used varying diagnostic criteria or included patients manifesting NODAT up to several years post-transplantation. These studies, which exclude patients with occult NODAT only identifiable via an OGTT likely report on a cohort of KTRs with a more severe disease phenotype in whom clinical NODAT is readily apparent. Thirdly, we cannot exclude that the unchanged survival in our NODAT cohort may reflect the intended benefit derived from a program of early screening and subsequent initiation of management strategies. Lastly, our study may be underpowered to detect an independent association between glycaemic status and mortality.

This study presents the strongest evidence to date in support of the use of OGTTs to identify KTRs with or at risk of NODAT. However, there are certain limitations to our study. Firstly, we evaluated a predominantly Caucasian population, and caution should therefore be applied when extrapolating to other ethnicities. Secondly, the post-transplant glycaemic status could not be adequately ascertained for some patients. Whilst these patients did not have clinical NODAT, we cannot exclude the presence of occult dysglycaemia that would have been detected by an OGTT. Additionally, we were not able to report on the presence of some factors known to contribute to development of NODAT, such as hyperlipidaemia and a family history of diabetes. However, whilst these factors are no doubt important considerations in the assessment of risk, their absence does not detract from the utility presented by an OGTT.

Our findings, whilst supporting those of Caillard’s data from the cyclosporine era (13), report on a significantly different cohort. Here, we demonstrate the utility of a pre-transplant OGTT in assessing the risk of future NODAT in the contemporary transplant era, in recipients of both deceased and living donor kidneys, treated predominantly with tacrolimus, mycophenolate and maintenance corticosteroids. Our findings clearly demonstrate the inadequacies of relying upon fasting glucose levels as a screening tool for abnormal glucose metabolism pre- and post-transplant. The benefits of performing an OGTT both prior to transplant, to inform risk of NODAT, and post-transplant, to detect NODAT and inform cardiovascular risk, are evident and in our opinion outweigh the modest associated economic costs and inconvenience. Ultimately, robust prospective trials are needed to determine whether various interventions, including choice of immunosuppression (40), alters the development of NODAT, major adverse cardiovascular events and mortality in high-risk individuals, such as those with pre-transplant IGT.

## Data Availability

Deindetified data pertaining to this study will be made available to investigators upon reasonable request and submission of a research plan of sufficient scientific merit. Requests to access the datasets should be directed to steve.chadban@health.nsw.gov.au.

## Abbreviations

2hPG, 2-h plasma glucose; ANZDATA, Australia and New Zealand Dialysis and Transplant Registry; BMI, body mass index; CNI, calcineurin inhibitor; eGFR, estimated glomerular filtration rate; ESKD, end-stage kidney disease; FPG, fasting plasma glucose; IFG, impaired fasting glucose; IGT, impaired glucose tolerance; KTRs, kidney transplant recipients; mTORi, mammalian target of rapamycin inhibitor; NODAT, new onset diabetes after transplant; OGTT, oral glucose tolerance test.
