# Supplementary material for: The Utility of Pre- and Post-Transplant Oral Glucose Tolerance Tests: Identifying Kidney Transplant Recipients With or at Risk of New Onset Diabetes After Transplant
Source: Transpl Int. 2022 Mar 17;35:10078. doi: 10.3389/ti.2022.10078 (PMC8967957; doi:10.3389/ti.2022.10078)
Supplement: Supplementary file 1 [file DataSheet1.DOCX]

|  | Haemodialysis | Peritoneal Dialysis | p |
| --- | --- | --- | --- |
|  | n = 370 | n = 136 |  |
| **Age (mean (SD))** | 48.30 (13.36) | 48.60 (13.93) | 0.825 |
| **BMI (mean (SD))** | 26.16 (5.17) | 25.67 (4.62) | 0.33 |
| **Prior Kidney Transplant (%)** | 60 (16.2) | 2 (1.5) | <0.001 |
| **Pre-Transplant OGTT Results** |  |  |  |
| FPG mmol/L (mean (SD)) | 4.81 (0.59) | 5.14 (0.66) | <0.001 |
| 2hPG mmol/L (mean (SD)) | 6.20 (1.78) | 6.64 (1.98) | 0.017 |
| **Glycaemic Status Pre-Transplant (%)** |  |  | <0.001 |
| IFG | 15 (4.1) | 23 (16.9) |  |
| IFG/IGT | 16 (4.3) | 10 (7.4) |  |
| IGT | 54 (14.6) | 27 (19.9) |  |
| Normoglycaemic | 285 (77.0) | 76 (55.9) |  |
| **Post-Transplant OGTT Results** |  |  |  |
| FPG mmol/L (mean (SD)) | 5.18 (0.67) | 5.12 (0.65) | 0.483 |
| 2hPG mmol/L (mean (SD)) | 8.20 (2.76) | 8.86 (3.14) | 0.08 |
| **Glycaemic Status Post-Transplant (%)** |  |  | 0.309 |
| IGT | 68 (18.4) | 32 (23.5) |  |
| Normoglycaemic | 109 (29.5) | 34 (25.0) |  |
| PTDM | 88 (23.8) | 38 (27.9) |  |
| Unknown | 105 (28.4) | 32 (23.5) |  |

Supplementary Table 1. Results of pre- and post-transplant oral glucose tolerance tests in patients undergoing renal replacement therapy prior to transplant.

|  | OGTT Performed | OGTT Not Performed | p |
| --- | --- | --- | --- |
|  | n = 358 | n = 156 |  |
| **Age (mean (SD))** | 46.1 ± 13.6 | 47.7 ±14.2 | 0.214 |
| **Gender** |  |  | 0.742 |
| Male (%) | 227 (63.4) | 102 (65.4) |  |
| Female (%) | 131 (36.6) | 54 (34.6) |  |
| **BMI (mean (SD))** | 25.7 ± 5.1) | 26.3 ± 4.9 | 0.246 |
| **Primary Renal Disease (%)** |  |  | 0.093 |
| Glomerulonephritis | 190 (53.1) | 65 (41.7) |  |
| Hypertension | 24 (6.7) | 9 (5.8) |  |
| Other | 65 (18.2) | 38 (24.4) |  |
| Polycystic Kidney Disease | 54 (15.1) | 26 (16.7) |  |
| Reflux Nephropathy/PUV | 25 (7.0) | 18 (11.5) |  |
| **Usual Nephrology care outside the transplant centre (%)** | 155 (43.3) | 101 (64.7) | <0.001 |
| **Smoking History (%)** | 113 (31.6) | 70 (44.9) | 0.005 |
| **Prior Vascular Disease† (%)** | 67 (18.7) | 26 (16.7) | 0.667 |
| **Living Donor (%)** | 208 (58.1) | 55 (35.3) | <0.001 |
| **Prior Kidney Transplant (%)** | 32 (8.9) | 25 (16.0) | 0.028 |
| **Early rejection (≤ 90 days post-transplant) (%)** | 51 (14.2) | 32 (20.5) | 0.1 |
| **Delayed Graft Function (%)** | 46 (12.8) | 38 (24.4) | 0.002 |
| **Pre-Transplant OGTT Results** |  |  |  |
| FPG mmol/L (mean (SD)) | 4.90 ± 0.58 | 4.81 ± 0.56 | 0.107 |
| 2hPG mmol/L (mean (SD)) | 6.10 ± 1.67 | 5.98 ± 1.80 | 0.46 |
| **Glycaemic Status Pre-Transplant (%)** |  |  | 0.72 |
| Normoglycaemic | 270 (75.4) | 122 (78.2) |  |
| IFG | 28 (7.8) | 9 (5.8) |  |
| IGT | 60 (16.8) | 25 (16.0) |  |

Supplementary Table 2. Characteristics of kidney transplant recipients without clinical NODAT, stratified by the presence or absence of a post-transplant OGTT result.

(†coronary artery disease, peripheral vascular disease, or cerebrovascular disease)

|  | crude OR (95% CI) | adjusted OR (95%CI) | P (Wald's Test) | P (LR-test) |
| --- | --- | --- | --- | --- |
| **Usual nephrology care outside the transplant centre** | 2.41 (1.63 - 3.55) | 2.42 (1.62 - 3.62) | < 0.001 | < 0.001 |
| **Current/Former smoker** | 1.76 (1.2 - 2.6) | 1.48 (0.98 - 2.21) | 0.059 | 0.06 |
| **Deceased donor** | 2.55 (1.72 - 3.76) | 2.25 (1.46 - 3.48 | < 0.001 | < 0.001 |
| **Delayed graft function** | 2.18 (1.35 - 3.53) | 1.42 (0.83 - 2.43) | 0.199 | 0.2 |

Supplementary Table 3. Covariates associated with the failure to obtain an oral glucose tolerance test post-transplant.
